# (Ethyl­enediamine-κ^2^ *N*,*N*)bis[2-(pyridin-2-yl-κ*N*)-1,3-imidazol-1-ido-κ*N* ^1^]cobalt(III) nitrate monohydrate

**DOI:** 10.1107/S1600536812032692

**Published:** 2012-08-08

**Authors:** Xiu-Ling Feng, Yu-Ping Zhang

**Affiliations:** a College of Chemistry and Chemical Engineering, Huaihua University, Huaihua 418008, People’s Republic of China; b Wuling Electric Power Group Corporation, Changsha 410000, People’s Republic of China

## Abstract

In the title compound, [Co(C_8_H_6_N_3_)_2_(C_2_H_8_N_2_)]NO_3_·H_2_O, the Co^III^ ion is coordinated by four N atoms from two 2-(pyridin-2-yl)-1,3-imidazol-1-ide ligands and two N atoms of ethyl­enediamine in a distorted octa­hedral geometry. In the crystal, classical N—H⋯N(O) and O—H⋯N(O) hydrogen bonds connect all the isolated components together to yield a three-dimensional structure.

## Related literature

For examples of metal–organic compounds containing the 2-(2-pyrid­yl)imidazole ligand, see: Dosser & Underhill (1972 ▶); Lan *et al.* (2008 ▶). For applications of these compounds, see: Carranza *et al.* (2009 ▶); Schott *et al.* (2011 ▶).
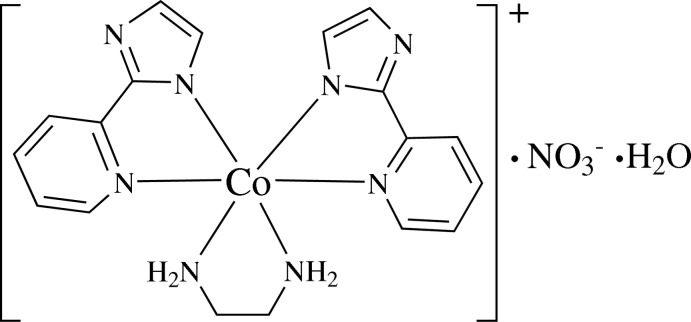


## Experimental

### 

#### Crystal data

[Co(C_8_H_6_N_3_)_2_(C_2_H_8_N_2_)]NO_3_·H_2_O
*M* *_r_* = 487.38
Triclinic, 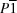

*a* = 8.6669 (5) Å
*b* = 11.0574 (8) Å
*c* = 12.5304 (10) Å
α = 76.133 (2)°
β = 75.672 (2)°
γ = 68.797 (1)°
*V* = 1069.62 (13) Å^3^
*Z* = 2
Mo *K*α radiation
μ = 0.85 mm^−1^
*T* = 298 K
0.45 × 0.38 × 0.30 mm

#### Data collection

Bruker SMART 1000 CCD diffractometer
Absorption correction: multi-scan (*SADABS*; Sheldrick, 1996 ▶) *T*_min_ = 0.699, *T*_max_ = 0.783
5376 measured reflections
3714 independent reflections
2907 reflections with *I* > 2σ(*I*)
*R*_int_ = 0.021

#### Refinement

*R*[*F* ^2^ > 2σ(*F* ^2^)] = 0.037
*wR*(*F* ^2^) = 0.110
*S* = 1.05
3714 reflections
289 parameters
?
Δρ_max_ = 0.32 e Å^−3^
Δρ_min_ = −0.42 e Å^−3^

### 

Data collection: *SMART* (Bruker, 2007 ▶); cell refinement: *SAINT* (Bruker, 2007 ▶); data reduction: *SAINT*; program(s) used to solve structure: *SHELXS97* (Sheldrick, 2008 ▶); program(s) used to refine structure: *SHELXL97* (Sheldrick, 2008 ▶); molecular graphics: *SHELXTL* (Sheldrick, 2008 ▶); software used to prepare material for publication: *SHELXTL*.

## Supplementary Material

Crystal structure: contains datablock(s) I, global. DOI: 10.1107/S1600536812032692/cv5321sup1.cif

Structure factors: contains datablock(s) I. DOI: 10.1107/S1600536812032692/cv5321Isup2.hkl

Additional supplementary materials:  crystallographic information; 3D view; checkCIF report

## Figures and Tables

**Table 1:** Hydrogen-bond geometry (Å, °)

*D*—H⋯*A*	*D*—H	H⋯*A*	*D*⋯*A*	*D*—H⋯*A*
N8—H8*B*⋯O1	0.90	2.16	2.922 (4)	142
N8—H8*A*⋯N5^i^	0.90	2.09	2.979 (3)	168
N7—H7*A*⋯N2^ii^	0.90	2.15	3.045 (3)	171
N7—H7*B*⋯O3^iii^	0.90	2.43	3.281 (5)	158
O4—H4*D*⋯N2^iii^	0.85	2.12	2.974 (7)	180
O4—H4*C*⋯O2^iv^	0.85	2.18	3.026 (7)	179

Symmetry codes: (i) 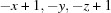; (ii) 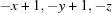; (iii) 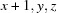; (iv) 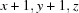.

**Fig. 1.**
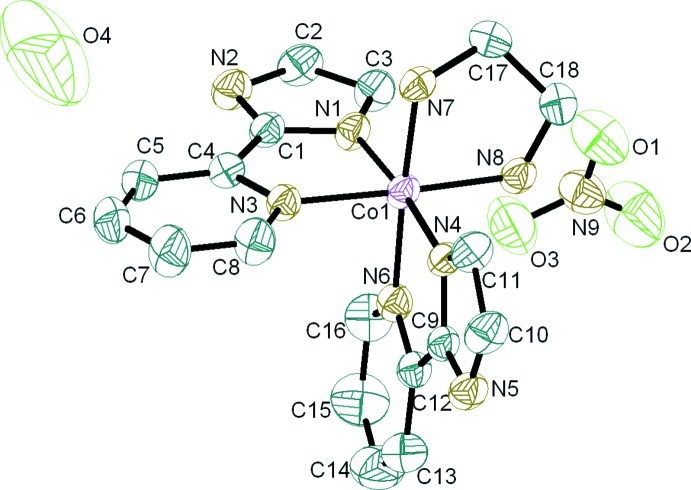
View of (I) showing the atom-numbering scheme. Displacement ellipsoids are drawn at the 50% probability level. H atoms have been omitted for clarity.

**Fig. 2.**
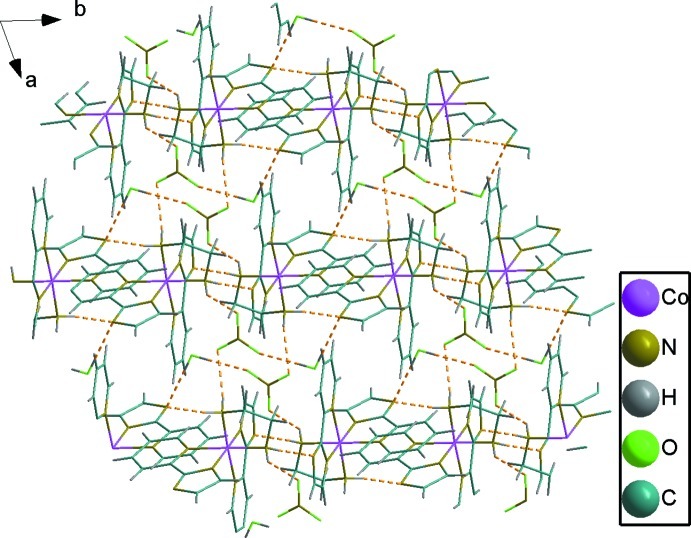
A portion of the crystal packing showing hydrogen bonds by dashed lines.

## supplementary crystallographic information

### Comment

Organometallic complexes with the 2-(2-pyridyl)imidazole ligand (Dosser & Underhill, 1972; Lan *et al.*, 2008) are intensively studied due to their magnetic properties (Carranza *et al.*, 2009; Schott *et al.*, 2011). Herewith we report the crystal structure of the title compound, (I) - a Co(III) complex with 2-(2-pyridyl)imidazolato ligands.

In (I) (Fig. 1), Co(III) ion is chelated by one ethylenediamine and two 2-(2-pyridyl)imidazolato ligands being coordinated by six N atoms in a distorted octahedral geometry. The bite angles of ethylenediamine and 2-(2-pyridyl)imidazolato chelate ligands to the cobalt atom are *ca* 84.72 (11)° and 89.02 (11)°, respectively. An extensive hydrogen-bonding network (Table 1) involving the N atoms of the ethylenediamine and 2-(2-pyridyl)imidazolato ligands, the water molecule and O atoms of nitrate anion interconnect all the isolated moieties together to yield a three-dimensional structure (Fig. 2).

### Experimental

An ethanol solution (7 ml) of 2-(2-pyridyl)imidazole (0.5 mmol) was slowly added to an aqueous solution (8 ml) of Co(NO_3_)_2_.6H_2_O (0.5 mmol) and ethylenediamine (2 mmol). Red block crystals were obtained after two months.

### Refinement

All H atoms were geometrically positioned (C—H = 0.93–0.97 Å; O—H = 0.85 Å; N—H = 0.90 Å) and treated as riding, with *U*_iso_(H) = 1.2*U*_eq_ of the parent atom.

### Crystal data

**Table:**

[Co(C_8_H_6_N_3_)_2_(C_2_H_8_N_2_)]NO_3_·H_2_O	*Z* = 2
*M**_r_* = 487.38	*F*(000) = 504
Triclinic, *P*1	*D*_x_ = 1.513 Mg m^−^^3^
Hall symbol: -P 1	Mo *K*α radiation, λ = 0.71073 Å
*a* = 8.6669 (5) Å	Cell parameters from 2876 reflections
*b* = 11.0574 (8) Å	θ = 2.6–28.0°
*c* = 12.5304 (10) Å	µ = 0.85 mm^−^^1^
α = 76.133 (2)°	*T* = 298 K
β = 75.672 (2)°	Block, red
γ = 68.797 (1)°	0.45 × 0.38 × 0.30 mm
*V* = 1069.62 (13) Å^3^	

### Data collection

**Table:**

Bruker SMART 1000 CCD diffractometer	3714 independent reflections
Radiation source: fine-focus sealed tube	2907 reflections with *I* > 2σ(*I*)
Graphite monochromator	*R*_int_ = 0.021
φ and ω scans	θ_max_ = 25.0°, θ_min_ = 2.6°
Absorption correction: multi-scan (*SADABS*; Sheldrick, 1996)	*h* = −7→10
*T*_min_ = 0.699, *T*_max_ = 0.783	*k* = −13→13
5376 measured reflections	*l* = −10→14

### Refinement

**Table:**

Refinement on *F*^2^	Primary atom site location: structure-invariant direct methods
Least-squares matrix: full	Secondary atom site location: difference Fourier map
*R*[*F*^2^ > 2σ(*F*^2^)] = 0.037	Hydrogen site location: inferred from neighbouring sites
*wR*(*F*^2^) = 0.110	*w* = 1/[σ^2^(*F*_o_^2^) + (0.0517*P*)^2^ + 0.5557*P*] where *P* = (*F*_o_^2^ + 2*F*_c_^2^)/3
*S* = 1.05	(Δ/σ)_max_ < 0.001
3714 reflections	Δρ_max_ = 0.32 e Å^−^^3^
289 parameters	Δρ_min_ = −0.42 e Å^−^^3^
0 restraints	

### Special details

**Table:**

Geometry. All e.s.d.'s (except the e.s.d. in the dihedral angle between two l.s. planes) are estimated using the full covariance matrix. The cell e.s.d.'s are taken into account individually in the estimation of e.s.d.'s in distances, angles and torsion angles; correlations between e.s.d.'s in cell parameters are only used when they are defined by crystal symmetry. An approximate (isotropic) treatment of cell e.s.d.'s is used for estimating e.s.d.'s involving l.s. planes.
Refinement. Refinement of *F*^2^ against ALL reflections. The weighted *R*-factor *wR* and goodness of fit *S* are based on *F*^2^, conventional *R*-factors *R* are based on *F*, with *F* set to zero for negative *F*^2^. The threshold expression of *F*^2^ > σ(*F*^2^) is used only for calculating *R*-factors(gt) *etc*. and is not relevant to the choice of reflections for refinement. *R*-factors based on *F*^2^ are statistically about twice as large as those based on *F*, and *R*-factors based on ALL data will be even larger.

### Fractional atomic coordinates and isotropic or equivalent isotropic displacement parameters (Å^2^)

**Table:**

	*x*	*y*	*z*	*U*_iso_*/*U*_eq_	
Co1	0.49327 (4)	0.24908 (3)	0.25014 (3)	0.03109 (14)	
N1	0.3734 (3)	0.3487 (2)	0.13063 (18)	0.0349 (5)	
N2	0.2556 (3)	0.5498 (2)	0.03542 (19)	0.0427 (6)	
N3	0.4925 (3)	0.4242 (2)	0.26227 (18)	0.0334 (5)	
N4	0.5997 (3)	0.1632 (2)	0.37782 (17)	0.0318 (5)	
N5	0.5660 (3)	0.1121 (2)	0.56550 (18)	0.0413 (6)	
N6	0.2860 (3)	0.2713 (2)	0.36447 (19)	0.0353 (5)	
N7	0.7032 (3)	0.2170 (2)	0.14273 (18)	0.0373 (5)	
H7A	0.7057	0.2916	0.0949	0.045*	
H7B	0.7911	0.1895	0.1785	0.045*	
N8	0.4899 (3)	0.0816 (2)	0.23036 (18)	0.0364 (5)	
H8A	0.4718	0.0319	0.2975	0.044*	
H8B	0.4055	0.0949	0.1946	0.044*	
N9	0.1402 (4)	0.0294 (3)	0.1721 (2)	0.0530 (7)	
O1	0.2907 (3)	−0.0084 (3)	0.1286 (2)	0.0716 (7)	
O2	0.0499 (4)	−0.0394 (3)	0.1852 (3)	0.1010 (11)	
O3	0.0786 (4)	0.1364 (3)	0.2036 (3)	0.1008 (11)	
O4	0.9518 (6)	0.7421 (6)	0.1433 (6)	0.251 (4)	
H4C	0.9804	0.8031	0.1547	0.301*	
H4D	1.0388	0.6873	0.1124	0.301*	
C1	0.3419 (3)	0.4799 (3)	0.1174 (2)	0.0342 (6)	
C2	0.2299 (4)	0.4556 (3)	−0.0051 (2)	0.0454 (7)	
H2	0.1728	0.4724	−0.0636	0.054*	
C3	0.3003 (4)	0.3323 (3)	0.0528 (2)	0.0431 (7)	
H3	0.2983	0.2531	0.0409	0.052*	
C4	0.4052 (3)	0.5251 (3)	0.1918 (2)	0.0353 (6)	
C5	0.3797 (4)	0.6549 (3)	0.1971 (2)	0.0454 (7)	
H5	0.3159	0.7230	0.1502	0.055*	
C6	0.4492 (5)	0.6817 (3)	0.2719 (3)	0.0553 (9)	
H6	0.4346	0.7682	0.2757	0.066*	
C7	0.5412 (5)	0.5790 (3)	0.3417 (3)	0.0585 (9)	
H7	0.5901	0.5955	0.3926	0.070*	
C8	0.5597 (4)	0.4522 (3)	0.3350 (3)	0.0476 (8)	
H8	0.6210	0.3834	0.3826	0.057*	
C9	0.4922 (3)	0.1662 (3)	0.4763 (2)	0.0336 (6)	
C10	0.7323 (4)	0.0724 (3)	0.5200 (2)	0.0457 (7)	
H10	0.8187	0.0298	0.5609	0.055*	
C11	0.7555 (4)	0.1034 (3)	0.4057 (2)	0.0416 (7)	
H11	0.8578	0.0869	0.3566	0.050*	
C12	0.3150 (4)	0.2280 (3)	0.4713 (2)	0.0363 (6)	
C13	0.1848 (4)	0.2462 (3)	0.5620 (3)	0.0535 (8)	
H13	0.2072	0.2190	0.6344	0.064*	
C14	0.0221 (5)	0.3050 (4)	0.5436 (3)	0.0677 (10)	
H14	−0.0673	0.3173	0.6034	0.081*	
C15	−0.0066 (4)	0.3452 (4)	0.4357 (3)	0.0690 (11)	
H15	−0.1162	0.3845	0.4222	0.083*	
C16	0.1268 (4)	0.3275 (3)	0.3472 (3)	0.0518 (8)	
H16	0.1058	0.3550	0.2745	0.062*	
C17	0.7132 (4)	0.1148 (3)	0.0810 (2)	0.0495 (8)	
H17A	0.8282	0.0759	0.0453	0.059*	
H17B	0.6434	0.1531	0.0239	0.059*	
C18	0.6521 (4)	0.0123 (3)	0.1648 (3)	0.0520 (8)	
H18A	0.6369	−0.0485	0.1269	0.062*	
H18B	0.7332	−0.0371	0.2136	0.062*	

### Atomic displacement parameters (Å^2^)

**Table:**

	*U*^11^	*U*^22^	*U*^33^	*U*^12^	*U*^13^	*U*^23^
Co1	0.0394 (2)	0.0299 (2)	0.0266 (2)	−0.01405 (16)	−0.01009 (15)	−0.00071 (14)
N1	0.0443 (13)	0.0330 (12)	0.0328 (12)	−0.0181 (11)	−0.0128 (10)	−0.0003 (10)
N2	0.0461 (14)	0.0413 (14)	0.0389 (13)	−0.0121 (11)	−0.0145 (11)	0.0003 (11)
N3	0.0393 (12)	0.0316 (12)	0.0321 (12)	−0.0134 (10)	−0.0094 (10)	−0.0037 (10)
N4	0.0398 (12)	0.0306 (12)	0.0274 (11)	−0.0154 (10)	−0.0070 (9)	−0.0015 (9)
N5	0.0557 (16)	0.0416 (14)	0.0306 (12)	−0.0203 (12)	−0.0124 (11)	−0.0010 (10)
N6	0.0393 (13)	0.0341 (12)	0.0354 (12)	−0.0137 (10)	−0.0096 (10)	−0.0052 (10)
N7	0.0456 (13)	0.0338 (12)	0.0305 (12)	−0.0138 (10)	−0.0078 (10)	0.0009 (10)
N8	0.0496 (14)	0.0357 (13)	0.0281 (12)	−0.0188 (11)	−0.0104 (10)	−0.0013 (10)
N9	0.0548 (18)	0.0607 (18)	0.0509 (16)	−0.0207 (15)	−0.0092 (13)	−0.0194 (14)
O1	0.0454 (14)	0.0897 (19)	0.0888 (19)	−0.0163 (13)	−0.0105 (13)	−0.0415 (16)
O2	0.077 (2)	0.112 (3)	0.142 (3)	−0.0543 (19)	0.0104 (19)	−0.064 (2)
O3	0.100 (2)	0.074 (2)	0.128 (3)	−0.0427 (18)	0.038 (2)	−0.052 (2)
O4	0.126 (4)	0.214 (6)	0.389 (10)	−0.007 (4)	0.033 (5)	−0.164 (7)
C1	0.0381 (15)	0.0319 (14)	0.0311 (14)	−0.0121 (12)	−0.0071 (11)	−0.0001 (11)
C2	0.0497 (17)	0.0535 (19)	0.0367 (16)	−0.0185 (15)	−0.0190 (13)	0.0005 (14)
C3	0.0534 (18)	0.0451 (17)	0.0393 (16)	−0.0229 (15)	−0.0169 (14)	−0.0026 (13)
C4	0.0375 (15)	0.0346 (15)	0.0324 (14)	−0.0123 (12)	−0.0041 (11)	−0.0039 (12)
C5	0.0570 (19)	0.0350 (16)	0.0419 (17)	−0.0139 (14)	−0.0082 (14)	−0.0041 (13)
C6	0.080 (2)	0.0385 (18)	0.054 (2)	−0.0238 (17)	−0.0104 (18)	−0.0120 (15)
C7	0.085 (3)	0.052 (2)	0.058 (2)	−0.0348 (19)	−0.0285 (19)	−0.0100 (17)
C8	0.0576 (19)	0.0460 (18)	0.0496 (18)	−0.0215 (15)	−0.0234 (15)	−0.0052 (14)
C9	0.0472 (16)	0.0294 (14)	0.0289 (14)	−0.0177 (12)	−0.0074 (12)	−0.0043 (11)
C10	0.0543 (19)	0.0497 (18)	0.0370 (16)	−0.0187 (15)	−0.0223 (14)	0.0038 (14)
C11	0.0404 (16)	0.0447 (17)	0.0398 (16)	−0.0138 (13)	−0.0132 (13)	−0.0004 (13)
C12	0.0458 (16)	0.0331 (15)	0.0338 (15)	−0.0177 (13)	−0.0056 (12)	−0.0064 (12)
C13	0.056 (2)	0.056 (2)	0.0438 (18)	−0.0184 (16)	−0.0002 (15)	−0.0083 (15)
C14	0.052 (2)	0.079 (3)	0.063 (2)	−0.0199 (19)	0.0076 (18)	−0.016 (2)
C15	0.0379 (18)	0.086 (3)	0.080 (3)	−0.0145 (18)	−0.0102 (18)	−0.016 (2)
C16	0.0452 (18)	0.059 (2)	0.055 (2)	−0.0163 (16)	−0.0173 (15)	−0.0084 (16)
C17	0.061 (2)	0.0477 (18)	0.0366 (16)	−0.0160 (16)	−0.0009 (14)	−0.0126 (14)
C18	0.069 (2)	0.0374 (17)	0.0482 (19)	−0.0168 (15)	−0.0047 (16)	−0.0100 (14)

### Geometric parameters (Å, º)

**Table:**

Co1—N4	1.919 (2)	C1—C4	1.447 (4)
Co1—N1	1.920 (2)	C2—C3	1.378 (4)
Co1—N8	1.938 (2)	C2—H2	0.9300
Co1—N7	1.949 (2)	C3—H3	0.9300
Co1—N3	1.976 (2)	C4—C5	1.387 (4)
Co1—N6	1.979 (2)	C5—C6	1.369 (4)
N1—C1	1.352 (3)	C5—H5	0.9300
N1—C3	1.364 (3)	C6—C7	1.380 (5)
N2—C1	1.339 (3)	C6—H6	0.9300
N2—C2	1.367 (4)	C7—C8	1.373 (4)
N3—C8	1.340 (4)	C7—H7	0.9300
N3—C4	1.358 (3)	C8—H8	0.9300
N4—C9	1.349 (3)	C9—C12	1.448 (4)
N4—C11	1.363 (3)	C10—C11	1.372 (4)
N5—C9	1.331 (3)	C10—H10	0.9300
N5—C10	1.361 (4)	C11—H11	0.9300
N6—C16	1.341 (4)	C12—C13	1.384 (4)
N6—C12	1.363 (3)	C13—C14	1.375 (5)
N7—C17	1.482 (4)	C13—H13	0.9300
N7—H7A	0.9000	C14—C15	1.373 (5)
N7—H7B	0.9000	C14—H14	0.9300
N8—C18	1.480 (4)	C15—C16	1.383 (5)
N8—H8A	0.9000	C15—H15	0.9300
N8—H8B	0.9000	C16—H16	0.9300
N9—O3	1.227 (4)	C17—C18	1.502 (4)
N9—O2	1.235 (4)	C17—H17A	0.9700
N9—O1	1.239 (3)	C17—H17B	0.9700
O4—H4C	0.8500	C18—H18A	0.9700
O4—H4D	0.8500	C18—H18B	0.9700
			
N4—Co1—N1	174.33 (9)	C2—C3—H3	126.5
N4—Co1—N8	89.92 (9)	N3—C4—C5	121.3 (3)
N1—Co1—N8	94.32 (9)	N3—C4—C1	112.4 (2)
N4—Co1—N7	94.58 (9)	C5—C4—C1	126.3 (3)
N1—Co1—N7	89.45 (10)	C6—C5—C4	119.3 (3)
N8—Co1—N7	86.37 (9)	C6—C5—H5	120.3
N4—Co1—N3	93.23 (9)	C4—C5—H5	120.3
N1—Co1—N3	82.56 (9)	C5—C6—C7	119.3 (3)
N8—Co1—N3	176.82 (9)	C5—C6—H6	120.3
N7—Co1—N3	92.92 (9)	C7—C6—H6	120.3
N4—Co1—N6	82.67 (9)	C8—C7—C6	119.1 (3)
N1—Co1—N6	93.44 (9)	C8—C7—H7	120.4
N8—Co1—N6	91.62 (9)	C6—C7—H7	120.4
N7—Co1—N6	176.60 (9)	N3—C8—C7	122.3 (3)
N3—Co1—N6	89.24 (9)	N3—C8—H8	118.8
C1—N1—C3	105.2 (2)	C7—C8—H8	118.8
C1—N1—Co1	113.99 (18)	N5—C9—N4	114.4 (2)
C3—N1—Co1	140.8 (2)	N5—C9—C12	129.0 (2)
C1—N2—C2	103.1 (2)	N4—C9—C12	116.6 (2)
C8—N3—C4	118.6 (2)	N5—C10—C11	110.9 (3)
C8—N3—Co1	127.1 (2)	N5—C10—H10	124.5
C4—N3—Co1	114.23 (18)	C11—C10—H10	124.5
C9—N4—C11	104.8 (2)	N4—C11—C10	106.8 (3)
C9—N4—Co1	114.15 (18)	N4—C11—H11	126.6
C11—N4—Co1	140.85 (19)	C10—C11—H11	126.6
C9—N5—C10	103.0 (2)	N6—C12—C13	121.5 (3)
C16—N6—C12	119.0 (3)	N6—C12—C9	112.5 (2)
C16—N6—Co1	127.1 (2)	C13—C12—C9	126.0 (3)
C12—N6—Co1	113.85 (18)	C14—C13—C12	119.1 (3)
C17—N7—Co1	108.11 (18)	C14—C13—H13	120.4
C17—N7—H7A	110.1	C12—C13—H13	120.4
Co1—N7—H7A	110.1	C15—C14—C13	119.0 (3)
C17—N7—H7B	110.1	C15—C14—H14	120.5
Co1—N7—H7B	110.1	C13—C14—H14	120.5
H7A—N7—H7B	108.4	C14—C15—C16	120.2 (3)
C18—N8—Co1	109.97 (18)	C14—C15—H15	119.9
C18—N8—H8A	109.7	C16—C15—H15	119.9
Co1—N8—H8A	109.7	N6—C16—C15	121.1 (3)
C18—N8—H8B	109.7	N6—C16—H16	119.5
Co1—N8—H8B	109.7	C15—C16—H16	119.5
H8A—N8—H8B	108.2	N7—C17—C18	107.1 (2)
O3—N9—O2	118.7 (3)	N7—C17—H17A	110.3
O3—N9—O1	120.1 (3)	C18—C17—H17A	110.3
O2—N9—O1	121.2 (3)	N7—C17—H17B	110.3
H4C—O4—H4D	108.4	C18—C17—H17B	110.3
N2—C1—N1	114.1 (2)	H17A—C17—H17B	108.5
N2—C1—C4	129.3 (2)	N8—C18—C17	107.4 (2)
N1—C1—C4	116.6 (2)	N8—C18—H18A	110.2
N2—C2—C3	110.6 (3)	C17—C18—H18A	110.2
N2—C2—H2	124.7	N8—C18—H18B	110.2
C3—C2—H2	124.7	C17—C18—H18B	110.2
N1—C3—C2	107.0 (3)	H18A—C18—H18B	108.5
N1—C3—H3	126.5		
			
N4—Co1—N1—C1	39.5 (10)	C3—N1—C1—N2	−0.9 (3)
N8—Co1—N1—C1	177.82 (19)	Co1—N1—C1—N2	−178.86 (18)
N7—Co1—N1—C1	−95.86 (19)	C3—N1—C1—C4	179.4 (2)
N3—Co1—N1—C1	−2.85 (18)	Co1—N1—C1—C4	1.4 (3)
N6—Co1—N1—C1	85.94 (19)	C1—N2—C2—C3	0.0 (3)
N4—Co1—N1—C3	−137.4 (8)	C1—N1—C3—C2	0.9 (3)
N8—Co1—N1—C3	0.9 (3)	Co1—N1—C3—C2	177.9 (2)
N7—Co1—N1—C3	87.3 (3)	N2—C2—C3—N1	−0.6 (3)
N3—Co1—N1—C3	−179.7 (3)	C8—N3—C4—C5	−2.5 (4)
N6—Co1—N1—C3	−91.0 (3)	Co1—N3—C4—C5	174.0 (2)
N4—Co1—N3—C8	4.0 (3)	C8—N3—C4—C1	179.3 (2)
N1—Co1—N3—C8	−179.9 (3)	Co1—N3—C4—C1	−4.2 (3)
N8—Co1—N3—C8	−167.7 (15)	N2—C1—C4—N3	−177.8 (3)
N7—Co1—N3—C8	−90.8 (3)	N1—C1—C4—N3	1.9 (3)
N6—Co1—N3—C8	86.6 (2)	N2—C1—C4—C5	4.1 (5)
N4—Co1—N3—C4	−172.22 (19)	N1—C1—C4—C5	−176.2 (3)
N1—Co1—N3—C4	3.96 (18)	N3—C4—C5—C6	2.5 (4)
N8—Co1—N3—C4	16.1 (17)	C1—C4—C5—C6	−179.6 (3)
N7—Co1—N3—C4	93.02 (19)	C4—C5—C6—C7	−1.0 (5)
N6—Co1—N3—C4	−89.61 (19)	C5—C6—C7—C8	−0.5 (5)
N1—Co1—N4—C9	43.2 (10)	C4—N3—C8—C7	1.0 (5)
N8—Co1—N4—C9	−95.29 (19)	Co1—N3—C8—C7	−175.1 (2)
N7—Co1—N4—C9	178.36 (18)	C6—C7—C8—N3	0.5 (5)
N3—Co1—N4—C9	85.17 (19)	C10—N5—C9—N4	0.2 (3)
N6—Co1—N4—C9	−3.64 (18)	C10—N5—C9—C12	−179.1 (3)
N1—Co1—N4—C11	−131.4 (8)	C11—N4—C9—N5	−0.6 (3)
N8—Co1—N4—C11	90.1 (3)	Co1—N4—C9—N5	−177.08 (17)
N7—Co1—N4—C11	3.8 (3)	C11—N4—C9—C12	178.8 (2)
N3—Co1—N4—C11	−89.4 (3)	Co1—N4—C9—C12	2.3 (3)
N6—Co1—N4—C11	−178.3 (3)	C9—N5—C10—C11	0.3 (3)
N4—Co1—N6—C16	−178.3 (3)	C9—N4—C11—C10	0.7 (3)
N1—Co1—N6—C16	5.8 (3)	Co1—N4—C11—C10	175.6 (2)
N8—Co1—N6—C16	−88.6 (3)	N5—C10—C11—N4	−0.6 (3)
N7—Co1—N6—C16	−142.2 (15)	C16—N6—C12—C13	−3.0 (4)
N3—Co1—N6—C16	88.3 (3)	Co1—N6—C12—C13	174.4 (2)
N4—Co1—N6—C12	4.48 (18)	C16—N6—C12—C9	178.2 (2)
N1—Co1—N6—C12	−171.38 (19)	Co1—N6—C12—C9	−4.3 (3)
N8—Co1—N6—C12	94.19 (19)	N5—C9—C12—N6	−179.3 (2)
N7—Co1—N6—C12	40.5 (16)	N4—C9—C12—N6	1.4 (3)
N3—Co1—N6—C12	−88.87 (19)	N5—C9—C12—C13	2.0 (5)
N4—Co1—N7—C17	107.72 (18)	N4—C9—C12—C13	−177.3 (3)
N1—Co1—N7—C17	−76.28 (19)	N6—C12—C13—C14	2.4 (5)
N8—Co1—N7—C17	18.09 (18)	C9—C12—C13—C14	−179.0 (3)
N3—Co1—N7—C17	−158.80 (18)	C12—C13—C14—C15	−0.6 (6)
N6—Co1—N7—C17	71.9 (16)	C13—C14—C15—C16	−0.5 (6)
N4—Co1—N8—C18	−85.02 (19)	C12—N6—C16—C15	1.9 (5)
N1—Co1—N8—C18	98.7 (2)	Co1—N6—C16—C15	−175.2 (3)
N7—Co1—N8—C18	9.57 (19)	C14—C15—C16—N6	−0.2 (6)
N3—Co1—N8—C18	86.7 (16)	Co1—N7—C17—C18	−41.4 (3)
N6—Co1—N8—C18	−167.69 (19)	Co1—N8—C18—C17	−34.7 (3)
C2—N2—C1—N1	0.5 (3)	N7—C17—C18—N8	49.6 (3)
C2—N2—C1—C4	−179.8 (3)		

### Hydrogen-bond geometry (Å, º)

**Table:**

*D*—H···*A*	*D*—H	H···*A*	*D*···*A*	*D*—H···*A*
N8—H8*B*···O1	0.90	2.16	2.922 (4)	142
N8—H8*A*···N5^i^	0.90	2.09	2.979 (3)	168
N7—H7*A*···N2^ii^	0.90	2.15	3.045 (3)	171
N7—H7*B*···O3^iii^	0.90	2.43	3.281 (5)	158
O4—H4*D*···N2^iii^	0.85	2.12	2.974 (7)	180
O4—H4*C*···O2^iv^	0.85	2.18	3.026 (7)	179

Symmetry codes: (i) −*x*+1, −*y*, −*z*+1; (ii) −*x*+1, −*y*+1, −*z*; (iii) *x*+1, *y*, *z*; (iv) *x*+1, *y*+1, *z*.
